# Short Term (14 Days) Consumption of Insoluble Wheat Bran Fibre-Containing Breakfast Cereals Improves Subjective Digestive Feelings, General Wellbeing and Bowel Function in a Dose Dependent Manner

**DOI:** 10.3390/nu5041436

**Published:** 2013-04-22

**Authors:** Clare L. Lawton, Jenny Walton, Alexa Hoyland, Elaine Howarth, Peter Allan, David Chesters, Louise Dye

**Affiliations:** 1 Human Appetite Research Unit, Institute of Psychological Sciences, University of Leeds, Leeds LS2 9JT, UK; E-Mail: l.dye@leeds.ac.uk; 2 The Kellogg Company, The Kellogg Building, Talbot Road, Manchester M16 0PU, UK; E-Mails: jenny.walton@kellogg.com (J.W.); alexa.hoyland@kellogg.com (A.H.); 3 Intertek CRS, Unit 6, Capenhurst Technology Park, Capenhurst, Cheshire CH1 6EH, UK; E-Mails: elaine.howarth@intertek.com (E.H.); peter.allan@intertek.com (P.A.); david.chesters@intertek.com (D.C.)

**Keywords:** dietary fibre, wheat bran, breakfast cereal, digestive health, bloating, bowel function, wellbeing

## Abstract

This study investigated whether increasing insoluble (predominantly wheat bran) fibre over 14 days improves subjective digestive feelings, general wellbeing and bowel function. A single centre, multi-site, open, within subjects design with a 14 day non-intervention (baseline) monitoring period followed by a 14 day fibre consumption (intervention) period was performed. 153 low fibre consumers (<15 g/day AOAC 985.29) completed a daily symptom diary for 14 days after which they consumed one bowl of ready-to-eat breakfast cereal containing at least 5.4 g fibre (3.5 g from wheat bran) for 14 days and completed a daily symptom diary. Significant improvements were demonstrated in subjective perception of bowel function (e.g., ease of defecation) and digestive feelings (bloating, constipation, feeling sluggish and digestive discomfort). Significant improvements were also found in subjective perception of general wellbeing (feeling less fat, more mentally alert, slim, happy and energetic whilst experiencing less stress, mental and physical tiredness, difficulty concentrating and fewer headaches). In general, improvements in study outcomes increased with increasing cereal/fibre consumption. However, consuming an additional minimum 5.4 g of fibre (3.5 g wheat bran) per day was shown to deliver measurable and significant benefits for digestive health, comfort and wellbeing. Encouraging consumption of relatively small amounts of wheat bran could also provide an effective method of increasing overall fibre consumption.

## 1. Introduction

Many people do not eat enough fibre. In the UK, the recommended dietary fibre intake is 18 g/day [1], based on non-starch polysaccharide (NSP) content (Englyst method). Furthermore, dietary recommendations in Europe range from 25 g/day to over 40 g/day based on the AOAC International Official Method 985.29. The average fibre intake of UK adults is currently about 13 g/day based on the Englyst method [2]. However, there is currently no accurate measurement of the AOAC International based average fibre intake in the UK and so it is difficult to compare fibre intakes across the EU and beyond. 

Higher fibre intake is associated with lower cardiovascular risk factors [3], healthier body weight [4], lower incidence of cancers of the breast [5] and colon [6], protection against diverticular disease [7] and most notably laxation [8]. Conversely, inadequate intake of insoluble fibre is associated with slow digestive transit and constipation, which can be accompanied by bloating and pain in the digestive system [9,10,11,12]. Survey data suggests that these symptoms are common in the general population. Van Kerkhoven *et al.* [10] reported that from a total of 5000 respondents in The Netherlands, 52% reported having had upper (43%) or lower (38%) gastrointestinal symptoms in the past four weeks. The most prevalent individual symptoms reported were flatulence (47%), abdominal rumbling (40%), bloating (37%), alternating solid and loose stools (31%), belching (25%) and postprandial fullness (25%). A similar internet based survey of 1215 UK adults [13] also found that 44% of people suffered from bloating, and 29% suffered from slow digestive transit and/or constipation. These symptoms are also commonly seen in clinical practice and are associated with a negative impact on general wellbeing and reduced quality of life [14,15]. Dietary interventions designed to reduce these negative symptoms, such as increasing intake of insoluble wheat bran fibre, which is minimally fermented in the large bowel, may, therefore, increase subjective wellbeing and quality of life through improved body image perception via decreased bloating.

A high fibre diet has been shown to be positively associated with increased wellbeing [16] and better physical and psychological health [17]. Some breakfast cereals are a good source of dietary fibre, with high fibre and wholegrain cereals contributing 11% of daily fibre intakes (based on NSP content in UK adults) [18]. Regular, breakfast consumption, especially consumption of high fibre cereals, is associated with fewer digestive problems such as constipation, bloating and abdominal/bowel pain [19,20,21,22] and better wellbeing (e.g., lower subjective scores of stress, anxiety, depression and emotional distress) [23]. Consumption of high fibre breakfast cereals might, therefore, impact on wellbeing by reducing digestive problems.

Although the benefits of increased fibre consumption for health and laxation are well accepted, few studies have actually explored the potential additional benefits of healthy and regular laxation such as improved psychological wellbeing and improved body image. Additionally, few studies have attempted to isolate the particular fibre responsible for any purported effects. However, in a recent study in healthy females with habitual low fibre intakes we demonstrated positive effects of a short-term (2-week) wheat bran fibre dietary intervention, using breakfast cereal and cereal based snacks, on both physiological and psychological wellbeing [24]. By week 2 of the intervention, almost all participants were consuming 8–14 g/day fibre (AOAC 985.29) from the study foods provided and had significantly increased their total daily fibre intake relative to baseline. In addition, daily wellbeing ratings indicated significant improvements in perceived stress, mental and physical tiredness, difficulty concentrating, hunger, craving unhealthy food and sluggishness with trends for reduction of feeling fat and bloating. In all cases, ratings were lower during the intervention period than at baseline. Furthermore, the quantity of fibre consumed was positively correlated with feeling slim and feeling content with body shape indicating dose related fibre benefits.

The aim of the present study was, therefore, to further investigate the effects of 2-weeks consumption of at least 5.4 g/day of fibre (AOAC 985.29, 3.5 g/day from wheat bran) from breakfast cereals on digestive, bowel function and wellbeing parameters in healthy habitual low-fibre consumers. Breakfast cereals were chosen as the vehicle for fibre provision as there are few other foods which can provide a significant amount of fibre without changing eating behaviour considerably. The duration of the fibre intervention period was selected in order to determine whether fibre benefits could be perceived over a relatively short time period as shown by our previous study. It was hypothesised that increasing the fibre intake (especially intake of wheat bran fibre) of low-fibre consumers would improve their subjective ratings of digestive feelings, general wellbeing (including ratings of feeling fat and feeling slim) and bowel function in a dose dependent manner. The primary objective of this study was to determine the effect of daily consumption of wheat bran containing breakfast cereals over a 2-week intervention period on digestive discomfort parameters in healthy adults who regularly consume a low-fibre diet. Secondary objectives were to evaluate effects on general wellbeing and bowel function parameters and the potential dose-dependence of negative symptom relief with greater wheat bran fibre intake. For clarity, all subsequent references to fibre intake in this paper are based on the AOAC International Official Method 985.29.

## 2. Materials and Methods

### 2.1. Participants

Participants were recruited from the general public via the Intertek CRS Volunteer Database. Volunteers were provided with the participant information sheet and 204 were screened (Visit 1) in the Ellesmere Port and Manchester region of the UK. Participants were required to be males or females in good health, aged between 18 and 50 years, with a body mass index (BMI) between 18.5 and 30 kg/m^2^ inclusive and willing and able to consume provided breakfast cereals (in place of any usual breakfast cereals) as part of the study. The main inclusion criterion was average consumption of less than 15 g dietary fibre per day (based on the AOAC International Official Method 985.29). Fibre intake was initially assessed using the Dietary Instrument for Nutrition Education (DINE) questionnaire, which has been validated against a detailed 4-day diet record [25]. Since the DINE only permits classification into low, medium or high fibre intake categories, an additional fibre intake questionnaire, designed to yield an average fibre intake in g/day (Leeds Fibre Intake Questionnaire (LFIQ), [26]) was also employed. This questionnaire used a scoring system based on the AOAC 985.29 fibre content of common foods (g of fibre/portion). Fibre intake (g/day) was derived by summing the products of the frequency of each fibre containing food consumed over a 7 day period (e.g., bread, cereals, fruit and vegetables, *etc.*) by the fibre content (g) based on a standard UK portion size of the food [27]. A previous study in low-fibre consumers found a strong positive correlation between fibre intake (g/day) assessed using the LFIQ and that assessed from 7-day food diary records [26].

Potential participants were excluded on the basis of; pregnancy, lactation, surgery in the previous 6 months, concurrent participation in another study involving a nutritional investigational product, participation in another study involving nutritional products during the previous 4 weeks prior to the start of the study, prior colostomy surgery, severe constipation or other medically diagnosed bowel problem/medication likely to interfere with the evaluation, use of over the counter laxatives in the previous 3 months, use of pre/probiotics in the previous 4 weeks, diagnosed coeliac disease or significant health problems as listed in the study protocol. Of the 204 potential participants, 48 were identified as ineligible at screening. Hence 156 volunteers were eligible and provided written informed consent to participate in the study.

The study was approved by Maldon Consumer Healthcare Research Ethics Committee. Written informed consent was obtained from all participants prior to their inclusion in the study.

### 2.2. Study Design

This study conformed to a single centre, multi-site, open, within subjects pre-post design. A 14 day non-intervention (baseline, habitual diet) monitoring period was followed by a 14 day fibre consumption (intervention) period.

### 2.3. Study Procedure

**Figure 1:**
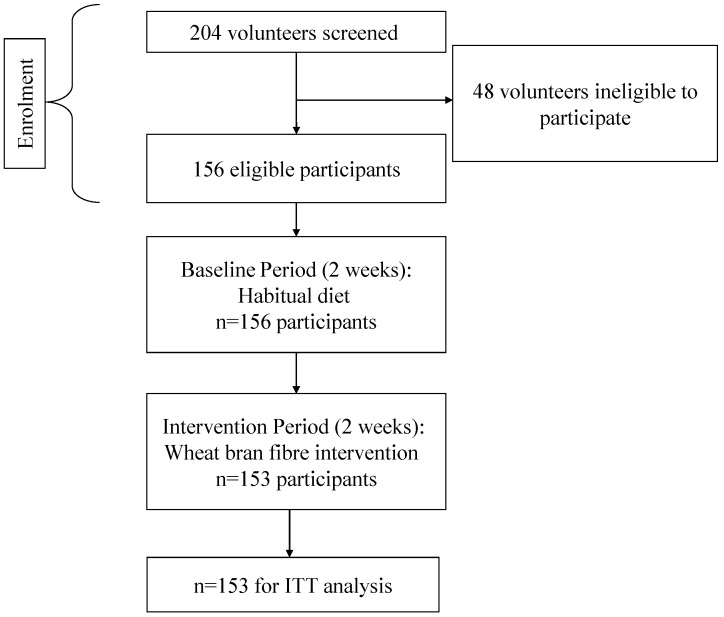
Consort diagram to show the flow of participants through each phase of the study.

Figure 1 shows the flow of participants through all phases of the study from screening onwards. Included participants were instructed to continue with their habitual diet and lifestyle and to complete a Digestive Wellbeing Questionnaire (DWQ) daily at a similar time each day (before retiring) for the next 14 days.

The DWQ was provided in an A5 size booklet and divided into three sections. The first section was designed to assess bowel habit, frequency and ease of defecation using the Bristol Stool Form Scale [28,29]. The following bowel function parameters were assessed after each bowel movement; stool type (from 1 to 7 according to the BSFS) and stool quantity (<average (0), average (1) or >average (2)), ease of “going to the toilet” (passing a stool) and satisfaction of bowel movement (after “going to the toilet”). Ease and satisfaction parameters were rated using 6 point Likert scales. For ease of defecation, scores ranged from 0 (very easy, effortless) to 5 (difficult, painful, force required). For satisfaction, scores ranged from 0 (dissatisfied, feels like there is more) to 5 (it’s all gone, I feel empty).

The second section measured digestive feelings using a 5 point Likert scale from 0 (none) to 4 (extreme) for each of the following descriptors; wind, constipation, indigestion, bloating, sluggish, digestive discomfort and pain in the digestive system. The final section measured general wellbeing using the same 5-point Likert scales to assess the following feelings; mental alertness, feeling slim, feeling happy, stress, mental tiredness, headaches, feeling energetic, feeling fat, difficulty concentrating and physical tiredness.

Following 14 days of continuing with their usual diet/lifestyle and completing the DWQ, participants returned to the research facility (Visit 2) with their completed booklet. All participants were obliged to receive the highest fibre containing breakfast cereal (Bran Shreds, 27 g fibre/100 g). However, they were invited to choose 3 additional products from a range of 7 commonly available, high fibre (9–27 g fibre/100 g) ready to eat breakfast cereals. The cereals were a range of flaked, shredded and wheat pillow cereals made from wheat bran. The fibre content of the provided cereals is shown in Table 1. Cereal was provided in opaque plastic liners with an ingredients list and product description only. Participants were also provided with a plastic scoop, to aid measurement, and instructed to consume at least one serving (2 × 100 mL scoop) per day, from any or a combination of the provided cereals. They were requested to ideally consume the cereals at breakfast, but if this was not convenient or desirable they were free to eat the cereal at any time of the day. Participants were then provided with a new DWQ, identical to the first but with an additional new section to record the type and number of scoops of each cereal consumed per day. For the next two weeks, they were asked to record their daily intake of the study breakfast cereal/s (number of scoops), complete the DWQ, and then return to the study site with their completed DWQs (Visit 3).

**Table 1:** Fibre content of the provided breakfast cereals.

Breakfast Cereal Type	Fibre (g) per 100 g	Weight (g) Cereal per Scoop	Fibre (g) per Scoop
Bran Shreds	27	36	9.72
Wheat Bran Flakes	15	30	4.50
Wheat Bran Flakes with Sultanas	13	30	3.90
Frosted Mini Wheats	9	30	2.70
Raisin Mini Wheats	9	30	2.70
Chocolate Wheat Bran Flakes	13	30	3.90
Apple & Fig Wheat Bran Flakes	15	40	6.00
Mean	14.4	32.3	4.8

The provided breakfast cereals varied considerably in volume and therefore the fibre content (all AOAC 985.29) per serving. However, the minimum daily intake (2 scoops), if consumed from the lowest fibre containing cereal, was 5.4 g of total fibre (3.5 g of which was fibre from wheat bran). This minimum intake was based on a study which showed that stool weight increased after 4 days of Kellogg’s All Bran consumption (providing 5.4 g of fibre/day) in normal healthy adults [30]. The actual daily intake of total and wheat bran fibre consumed from the study cereals was calculated for each participant (per day) from the number of scoops consumed in conjunction with the known fibre content per scoop (Table 1). Participants were instructed to refrain from eating other breakfast cereals or any other pre or probiotic products but to otherwise adhere to their usual diet throughout the study.

### 2.4. Statistical Analysis

All statistical analyses were conducted using SAS version 9.2 [31]. Data from 153 participants who completed both the 14 day non-intervention (baseline, habitual diet) monitoring period and the 14-day fibre consumption (intervention) period were analysed. The criteria for evaluation, was the comparison of change in Likert scale responses between the baseline and the fibre intervention period. Missing data were not imputed and were treated as missing for the statistical analysis.

Basic summary statistics were calculated for digestive feelings, general wellbeing and bowel function parameters during both the baseline and fibre intervention periods. Frequencies for each score level (response category), for each outcome variable, were also produced. The difference in mean symptom scores between the baseline and fibre intervention periods were compared using the Wilcoxon signed rank test.

The number of scoops of test cereal consumed per day (across all 7 cereals) was calculated for each participant and the data were split into 4 intake groups as shown below:

- Group 1 (*n* = 35): 2 scoops or fewer per day (*i.e.*, scoops ≤ 2);
- Group 2 (*n* = 52): more than 2, but no more than 2.5 scoops (*i.e.*, 2 *<* scoops ≤ 2.5);
- Group 3 (*n* = 29): more than 2.5, but no more than 3 scoops (*i.e.*, 2.5 *<* scoops ≤ 3);
- Group 4 (*n* = 37): more than 3 scoops (*i.e.*, scoops > 3).

The mean fibre (g) intake within each fibre group was compared using Analysis of Variance (ANOVA) whilst the mean symptom scores within each fibre intake group were compared using the Kruskal-Wallis test. *Post-hoc* tests were conducted to identify significant differences between fibre intake groups. Adjustments for possible differences due to the confounding variable (baseline score) were made using Analysis of Covariance (ANCOVA) when model assumptions were met. The ANCOVA assumption of homogeneity of regression slopes was not violated for scores of wind (flatulence), ease of defecation, mental alertness, feeling slim, feeling energetic and physical tiredness (*p*-value > 0.05 for the interaction between baseline score and group in each case).

Secondary analyses were also conducted on bloating data from participants who reported higher scores (≥3) at baseline. The Wilcoxon signed rank test was used to examine the shifts in the responses of the ordinal data between the baseline and fibre intervention periods. The proportions (percentage and number) of on-diagonal and off-diagonal responses were reported as:

- Decrease in symptoms (intervention response < baseline response, off diagonal response);
- No change (*i.e.*, intervention response = baseline response, on diagonal response);
- Increase in symptoms (*i.e.*, intervention response > baseline response, off diagonal response).

All statistical tests applied were two-sided and at the 5% significance level.

## 3. Results

### 3.1. Participant Characteristics

Of the 153 participants, there were 81 (52.9%) females and 72 (47.1%) males. Mean (SD) age was 33.7 (9.0) years. Mean (SD) BMI was 24.5 (3.0) kg/m^2^. The mean (SD) baseline total fibre intake assessed using the LFIQ was 10.5 (3.2) g/day (range: 2.8–15.4 g/day). 

### 3.2. Breakfast Cereal and Fibre Intake

During the 14 day intervention period, the number of scoops of breakfast cereal consumed per day, ranged from 2 to 5 scoops (mean = 2.62, SD = 0.68). Mean (SD) total fibre intake from the provided breakfast cereals was 13.9 (4.7) g/day (range: 7.8–31.6 g/day) and mean (SD) wheat bran fibre intake was 9.8 (3.3) g/day (range: 5.4–22.1 g/day). When participants were split by scoop intake Group, mean (SD) total fibre intake from the provided breakfast cereals was 10.7 (2.13) g/day for Group 1, 11.9 (2.82) g/day for Group 2, 14.2 (2.90) g/day for Group 3 and 19.9 (4.49) g/day for Group 4. There was a main effect of Group on fibre intake from the breakfast cereals (*p* < 0.0001). All scoop Groups differed significantly in terms of their fibre intake from the breakfast cereals with the exception of Groups 1 and 2.

### 3.3. Digestive Feelings

Table 2 shows the mean (SD) of the reported digestive feeling Likert scale scores (together with those for bowel function and general wellbeing) during both the 14 day non-intervention (baseline, habitual diet) monitoring period and the 14 day fibre consumption (intervention) period. Consumption of the high wheat bran fibre containing cereals led to significant improvements in the following digestive feelings; constipation, bloating, sluggish and digestive discomfort. Ratings of wind (flatulence) were, however significantly higher during the fibre intervention period.

**Table 2:** Digestive Feelings ^1^, Bowel Function ^2^ and General Wellbeing ^1^: Summary statisticsfor the daily Likert scale scores provided across each 14 day period (153 participants).

	Baseline Period (day 1 to 14)	Fibre Intervention Period (day 15 to 28)	Wilcoxon Signed Rank (S)	Significance (*p*-value)
*Digestive Feelings*				
Wind	1.11 ± 0.95	1.22 ± 1.04	54,109.5	<0.0001
Constipation	0.43 ± 0.80	0.36 ± 0.69	−19,153.0	0.0002
Indigestion	0.29 ± 0.61	0.27 ± 0.58	−4297.0	NS
Bloated	0.76 ± 0.97	0.57 ± 0.82	−67,846.0	<0.0001
Sluggish	0.66 ± 0.89	0.43 ± 0.71	−81,060.5	<0.0001
Digestive discomfort	0.46 ± 0.81	0.40 ± 0.72	−19,829.5	0.0004
Pain in the digestive system	0.27 ± 0.64	0.29 ± 0.63	5001.5	NS
*Bowel Function*				
Ease of defecation	1.29 ± 1.01	1.06 ± 0.90	−73,602.5	<0.0001
Satisfaction of bowel movement	2.34 ± 1.13	2.53 ± 1.07	64,513.5	<0.0001
Stool Type	3.49 ± 1.28	3.80 ± 1.14	84,255.5	<0.0001
Stool Quantity	0.84 ± 0.61	0.90 ± 0.62	35,567.0	<0.0001
*General Wellbeing*				
Mental alertness	1.91 ± 0.91	2.01 ± 0.88	37,017.0	<0.0001
Feeling slim	1.37 ± 1.07	1.57 ± 1.11	82,233.5	<0.0001
Feeling happy	2.07 ± 0.87	2.19 ± 0.86	54,553.5	<0.0001
Stress	0.99 ± 0.99	0.82 ± 0.92	−63,785.5	<0.0001
Mental tiredness	1.18 ± 1.0	0.97 ± 0.91	−92,963.5	<0.0001
Headache	0.41 ± 0.81	0.33 ± 0.68	−17,583.0	0.0005
Feeling energetic	1.61 ± 0.92	1.81 ± 0.91	88,172.5	<0.0001
Feeling fat	0.95 ± 1.11	0.74 ± 0.95	−65,848.0	<0.0001
Difficulty concentrating	0.91 ± 0.98	0.71 ± 0.82	−74,811.0	<0.0001
Physical tiredness	1.27 ± 1.06	0.98 ± 0.94	−131,389.0	<0.0001

Values are shown as mean ± standard deviation; ^1^ Digestive feeling and General wellbeing ratings: 0 = none, 1 = minimal, 2 = moderate, 3 = a lot/very, 4 = extreme; ^2^ See Table 3 for bowel parameter scoring key.

Table 3 shows the frequencies of the reported Likert scale scores (*i.e.*, the number of days on which a score in each category was reported) for each of the seven digestive feelings (together with those for bowel function and general wellbeing) assessed during both the 14 day non-intervention (baseline, habitual diet) monitoring period and the 14 day fibre consumption (intervention) period. These data show the shift in the distribution of the Likert scale scores across the score categories between the baseline and the fibre intervention period.

**Table 3:** Digestive feelings, Bowel Function and General Wellbeing: Frequency ^1^ (percent) of days of reporting of each level of the Likert scale scores across each 14 day period (153 participants).

	Baseline Period (day 1 to 14)	Fibre Intervention Period (day 15 to 28)
*Digestive Feelings*		
**Wind **		
None	645 (30.3)	622 (29.2)
Minimal	791 (37.2)	724 (34.0)
Moderate	503 (23.7)	514 (24.1)
A lot/Very	178 (8.4)	240 (11.2)
Extreme	9 (0.4)	32 (1.5)
**Constipation**		
None	1534 (72.3)	1585 (74.5)
Minimal	334 (15.8)	362 (17.0)
Moderate	191 (9.0)	147 (6.9)
A lot/Very	51 (2.4)	29 (1.4)
Extreme	11 (0.5)	5 (0.2)
**Indigestion**		
None	1650 (78.0)	1692 (79.5)
Minimal	336 (15.9)	314 (14.7)
Moderate	105 (4.9)	113 (5.3)
A lot/Very	23 (1.1)	11 (0.5)
Extreme	1 (0.1)	0 (0.0)
**Bloated**		
None	1137 (53.5)	1299 (61.0)
Minimal	517 (24.4)	512 (24.0)
Moderate	312 (14.7)	255 (12.0)
A lot/Very	146 (6.9)	61 (2.9)
Extreme	10 (0.5)	2 (0.1)
**Sluggish**		
None	1212 (57.0)	1458 (68.4)
Minimal	522 (24.6)	472 (22.2)
Moderate	295 (13.9)	166 (7.8)
A lot/Very	87 (4.1)	33 (1.5)
Extreme	8 (0.4)	1 (0.1)
**Digestive discomfort**		
None	1483 (70.0)	1533 (71.9)
Minimal	361 (17.0)	400 (18.8)
Moderate	207 (9.8)	150 (7.0)
A lot/Very	63 (3.0)	47 ( 2.2)
Extreme	5 (0.2)	1 (0.1)
**Pain in the digestive system**		
None	1735 (81.7)	1679 (78.8)
Minimal	249 (11.7)	311 (14.6)
Moderate	104 (4.9)	106 (5.1)
A lot/Very	32 (1.5)	31 (1.4)
Extreme	5 (0.2)	1 (0.1)
*Bowel Function*		
**Ease of defecation **		
0: Very easy, effortless	449 (24.3)	590 (30.4)
1: Fairly easy	679 (36.7)	757 (39.1)
2: Moderate, little effort required	491 (26.6)	485 (25.0)
3: Required effort	192 (10.5)	91 (4.7)
4: Difficult, straining required	36 (1.9)	15 ( 0.8)
5: Difficult, painful, force required	0 (0.0)	0 (0.0)
**Satisfaction of bowel movement**		
0: Dissatisfied, feels like there is more	100 (5.5)	69 (3.6)
1: Still feel like I need to go	317 (17.4)	234 (12.1)
2: No descriptor provided	602 (33.0)	639 (33.2)
3: No descriptor provided	458 (25.2)	574 (29.8)
4: Almost perfect	343 (18.9)	410 ( 21.3)
5: It’s all gone, I feel empty	0 (0.0)	0 (0.0)
**Stool Type**		
1: hard to pass	187 (7.7)	90 (3.4)
2: hard to pass	289 (12.1)	192 (7.3)
3: ideal consistency	700 (29.3)	678 (25.7)
4: ideal consistency	749 (31.3)	1073 (40.8)
5: Difficult to control	340 (14.2)	409 (5.6)
6: Difficult to control	99 (4.2)	168 (6.4)
7: Difficult to control	28 (1.2)	21 (0.8)
**Stool Quantity**		
0: less than average	668 (27.8)	659 (24.6)
1: average	1451 (60.6)	1618 (60.5)
2: more than average	277 (11.6)	397 (14.9)
*General Wellbeing*		
**Mental alertness**		
None	222 (10.4)	176 (8.3)
Minimal	273 (12.8)	234 (11.0)
Moderate	1157 (54.3)	1150 (54.3)
A lot/Very	432 (20.4)	506 (23.9)
Extreme	45 (2.1)	53 (2.5)
**Feeling slim**		
None	582 (27.4)	458 (21.6)
Minimal	515 (24.3)	513 (24.3)
Moderate	724 (34.1)	679 (32.1)
A lot/Very	262 (12.4)	411 (19.4)
Extreme	38 (1.8)	55 (2.6)
**Feeling happy**		
None	122 (5.8)	99 (4.7)
Minimal	301 (14.2)	237 (11.2)
Moderate	1089 (51.3)	1020 (48.2)
A lot/Very	537 (25.3)	675 (31.9)
Extreme	74 (3.4)	84 (4.0)
**Stress**		
None	822 (38.6)	974 (46.0)
Minimal	719 (33.8)	668 (31.6)
Moderate	412 (19.4)	365 (17.2)
A lot/Very	143 (6.7)	90 (4.3)
Extreme	32 (1.5)	18 (0.9)
**Mental tiredness**		
None	650 (30.6)	783 (37.0)
Minimal	677 (31.9)	713 (33.7)
Moderate	575 (27.1)	515 (24.4)
A lot/Very	207 (9.7)	100 (4.7)
Extreme	16 (0.7)	4 (0.2)
**Headache**		
None	1571 (74.1)	1628 (77.1)
Minimal	325 (15.3)	313 (14.8)
Moderate	143 (6.8)	131 (6.2)
A lot/Very	59 (2.8)	35 (1.7)
Extreme	21 (1.0)	4 (0.2)
**Feeling energetic**		
None	292 (3.7)	207 (9.8)
Minimal	559 (26.3)	444 (21.1)
Moderate	994 (46.8)	1048 (49.7)
A lot/Very	242 (11.4)	360 (17.1)
Extreme	38 (1.8)	49 (2.3)
**Feeling fat**		
None	1020 (48.1)	1134 (54.0)
Minimal	465 (21.9)	508 (24.2)
Moderate	404 (19.0)	331 (15.8)
A lot/Very	185 (8.7)	116 (5.5)
Extreme	49 (2.3)	10 (0.5)
**Difficulty concentrating**		
None	926 (43.6)	1051 (49.8)
Minimal	649 (30.5)	661 (31.3)
Moderate	404 (19.0)	355 (16.8)
A lot/Very	117 (5.5)	44 (2.1)
Extreme	30 (1.4)	0 (0.0)
**Physical tiredness**		
None	634 (29.7)	824 (39.0)
Minimal	592 (27.8)	634 (30.0)
Moderate	626 (29.4)	534 (25.2)
A lot/Very	248 (11.6)	118 (5.6)
Extreme	31 (1.5)	5 (0.2)

^1^ Maximum total frequency for each parameter is 153 participants × 14 days of recording = 2142, a lower total frequency is explained by missing data.

Table 4 shows the mean (SD) digestive feelings (together with those for bowel function and general wellbeing) according to cereal intake group (Groups 1–4). Analyses showed a main effect of Group for all symptoms (largest *p =* 0.024, Kruskal-Wallis χ*^2^* = 9.43, *df* = 3). Participants who consumed more than 3 scoops of cereal per day (Group 4) had significantly higher wind scores compared to those who consumed between 2 and 2.5 scoops per day (Group 2). In addition participants consuming more than 2 scoops of cereal per day (Groups 2–4) had significantly lower constipation, indigestion, bloated, sluggish and digestive discomfort scores compared to those who consumed 2 scoops or less of cereal per day (Group 1). Finally, participants who consumed between 2.5 and 3 scoops of cereal per day (Group 3) had significantly lower scores for pain in the digestive system compared to those who consumed 2.5 scoops or less per day (Groups 1–2).

Secondary analyses were conducted on data from participants who reported higher scores of bloating (score ≥ 3) at baseline. This was undertaken in order to evaluate whether those participants who reported a greater degree of bloating benefitted most from the fibre intervention. Previous empirical data [10,13] indicate that approximately 44% of the European population suffers from bloating. On this basis, it was estimated that 44% of the participants in the present study would report bloating. Table 3 shows that a similar proportion (46.5%) of participants in the present study reported feeling at least minimal bloating at baseline (scores ≥ 1). Hence the prevalence of bloating in the study sample was representative of that in the general Western population. 

**Table 4:** Fibre intake, Digestive feelings ^1^, Bowel function ^2^ and General wellbeing ^1^ according to Cereal Intake Group (153 participants) during the 14 day fibre intervention period. All mean values are unadjusted except where indicated.

Cereal Intake Group	Group 1	Group 2	Group 3	Group 4		
	Scoops ≤ 2 (*n* = 35)	2 *<* Scoops ≤ 2.5 (*n* = 52)	2.5 *<* Scoops ≤ 3 (*n* = 29)	Scoops > 3 (*n* = 37)		
	Mean ± SD	Mean ± SD	Mean ± SD	Mean ± SD	Critical Value ^3^	Significance (*p*-value)
Fibre Intake (g)	10.7 ± 2.13	11.9 ± 2.82	14.2 ± 2.90	19.9 ± 4.49	61.56	<0.0001
*Digestive Feelings*						
Wind	1.24 ± 1.10	1.13 ± 0.99	1.23 ± 1.00	1.31 ± 1.05	9.43	0.0241
*Adjusted Mean*	*1.26*	*1.14*	*1.22*	*1.30*	*3.05*	*0.0275*
Constipation	0.47 ± 0.81	0.30 ± 0.63	0.28 ± 0.59	0.39 ± 0.71	20.26	0.0002
Indigestion	0.41 ± 0.69	0.20 ± 0.50	0.26 ± 0.57	0.25 ± 0.55	41.23	<0.0001
Bloated	0.73 ± 0.90	0.57 ± 0.81	0.52 ± 0.77	0.47 ± 0.76	26.94	0.0001
Sluggish	0.51 ± 0.78	0.43 ± 0.70	0.33 ± 0.60	0.42 ± 0.71	12.11	0.0070
Digestive discomfort	0.49 ± 0.74	0.38 ± 0.71	0.35 ± 0.68	0.37 ± 0.74	19.98	0.0002
Pain in the digestive system	0.37 ± 0.66	0.30 ± 0.64	0.21 ± 0.55	0.28 ± 0.65	21.95	<0.0001
*Bowel Function*						
Ease of defecation	1.14 ± 0.90	1.10 ± 0.91	0.92 ± 0.83	1.06 ± 0.93	12.40	0.0061
*Adjusted Mean*	*1.09*	*1.08*	*0.90*	*1.09*	*4.13*	*0.0063*
Satisfaction of bowel movement	2.51 ± 1.09	2.55 ± 1.00	2.60 ± 1.09	2.47 ± 1.10	3.29	NS
Stool Type	3.48 ± 1.26	3.85 ± 1.12	3.93 ± 1.07	3.93 ± 1.05	43.82	<0.0001
Stool Quantity	0.85 ± 0.60	0.92 ± 0.60	0.90 ± 0.66	0.93 ± 0.63	5.95	NS
*Adjusted Mean*	*0.92*	*0.95*	*1.01*	*0.94*	*1.53*	*NS*
*General Wellbeing*						
Mental alertness	1.90 ± 0.83	2.08 ± 0.92	2.00 ± 0.94	1.90 ± 0.83	16.53	0.0009
*Adjusted Mean*	*1.92*	*2.05*	*2.07*	*1.99*	*3.55*	*0.0139*
Feeling slim	1.35 ± 1.08	1.68 ± 1.11	1.57± 1.13	1.62 ± 1.08	22.33	<0.0001
*Adjusted Mean*	*1.49*	*1.67*	*1.47*	*1.58*	*6.15*	*0.0004*
Feeling happy	2.15 ± 0.73	2.23 ± 0.84	2.06 ± 1.00	2.28 ± 0.88	16.29	0.0010
Stress	0.84 ± 0.97	0.88 ± 0.90	0.95 ± 1.04	0.63 ± 0.77	29.75	<0.0001
Mental tiredness	0.95 ± 0.91	1.04 ± 0.90	1.02 ± 0.91	0.87 ± 0.90	12.69	0.0053
Headache	0.32 ± 0.68	0.40 ± 0.73	0.29 ± 0.66	0.27 ± 0.62	15.30	0.0016
Feeling energetic	1.77 ± 0.81	1.79 ± 0.94	1.76 ± 0.97	1.91 ± 0.90	8.91	0.0305
*Adjusted Mean*	*1.75*	*1.82*	*1.75*	*1.89*	*3.01*	*0.0291*
Feeling fat	0.81 ± 1.02	0.84 ± 1.00	0.82 ± 0.90	0.48 ± 0.77	53.13	<0.0001
Difficulty concentrating	0.64 ± 0.79	0.73 ± 0.83	0.83 ± 0.84	0.66 ± 0.80	15.82	0.0012
Physical tiredness	0.93 ± 0.90	1.02 ± 0.94	1.06 ± 0.98	0.91 ± 0.95	9.01	0.0292
*Adjusted Mean*	*0.95*	*0.97*	*1.05*	*0.96*	*1.14*	*NS*

^1^ Digestive feeling and General wellbeing ratings: 0 = none, 1 = minimal, 2 = moderate, 3 = a lot/very, 4 = extreme; ^2^ See Table 3 for bowel parameter scoring key; ^3^ ANOVA for fibre intake, otherwise Kruskal-Wallis (χ^2^) for unadjusted means, ANCOVA (*F*) for adjusted means, *df* = 3 for all parameters.

Table 3, Table 5 (which shows the frequencies of the reported Likert scale scores when both the baseline and intervention period score was present within the same subject for the same day) show the improvement in the scores for “feeling bloated” from the baseline to the fibre intervention period (Wilcoxon signed rank *p* < 0.0001). Table 6 shows that for cases with data at both time points, 28.1% (594/2110) reported a decrease, 55.7% (1175/2110) reported no change and 16.2% (341/2110) reported an increase in feelings of “bloated” from the baseline to the fibre intervention period. In those cases with higher scores for feeling bloated (≥3) at baseline (*n* = 155), there was a significant improvement in the distribution of the scores from the baseline to the fibre intervention period (Wilcoxon signed rank test *p* < 0.0001). Table 6 shows that 89% (138/155) of these participants reported a decrease and 11% reported no change in feelings of “bloated” from the baseline to the fibre intervention period. Hence those participants who reported a greater degree of bloating at baseline benefitted most from the fibre intervention. A greater percentage of these participants reported a decrease in feelings of “bloated” in response to the fibre intervention compared to the percentage of the total sample reporting such a decrease (89% *vs.* 28.1%).

**Table 5:** Frequency of days of reporting of each level of the Likert scale scores for the digestive feeling “bloated” during each 14 day period, for participants with complete data at both time points. Frequencies are shown for participants with any “bloated” score at baseline (all scores) and for participants with higher scores (≥3) at baseline (extreme scores).

Bloated scores	0 None	1 Minimal	2 Moderate	3 A Lot/Very	4 Extreme	Total
All scores at baseline						
Baseline period	1131	514	310	145	10	2110
Fibre intervention period	1289	504	254	61	2	2110
Higher scores at baseline						
Baseline period				145	10	155
Fibre intervention period	50	49	38	17	1	155

**Table 6:** The shifts in scores of the digestive feeling “bloated” from the baseline to the fibre intervention period for all participants with data at both time points. Change ^1^ frequencies are shown for participants with any score at baseline (all scores) and for participants with higher scores (≥3) at baseline (extreme scores).

Bloated	Decrease in symptoms				No Change	Increase in symptoms				Total
Change in digestive feeling	−4	−3	−2	−1	0	+1	+2	+3	+4	
All scores at baseline										
Frequency	2	50	151	391	1175	250	78	13	0	2110
Higher scores at baseline										
Frequency	2	50	51	35	17	0	0	0	0	155

^1^ Change = Intervention response–Baseline response.

### 3.4. Bowel Function

Table 2 shows the mean (SD), and Table 3 shows the frequency (percentage), of the reported bowel function Likert scale scores during both the 14 day non-intervention (baseline, habitual diet) monitoring period and the 14 day fibre consumption (intervention) period. Consumption of the high wheat bran fibre containing cereals led to significant improvements in ease of defecation, satisfaction with bowel movement and stool type (Wilcoxon Signed Rank all *p* < 0.0001). These improvements occurred in conjunction with a significant increase in reported stool quantity (Wilcoxon Signed Rank *p* < 0.0001).

Table 4 shows the mean (SD) bowel function parameter scores according to cereal intake group (Groups 1–4). There was a significant difference between the fibre intake groups for ease of defecation (Kruskal-Wallis *p* = 0.0061) and stool type (Kruskal-Wallis *p* < 0.0001). There was no significant difference between fibre intake groups for satisfaction with bowel movement or stool quantity. On average, participants consuming between 2.5 and 3 scoops of cereal per day (Group 3) reported statistically significantly greater ease of defecation compared to participants who consumed 2.5 scoops or less per day (Groups 1 and 2) or more than 3 scoops of cereal per day (Group 4).

### 3.5. General Wellbeing

Table 2 shows the mean (SD), and Table 3 shows the frequency (percentage), of the reported general wellbeing Likert scale scores during both the 14 day non-intervention (baseline, habitual diet) monitoring period and the 14 day fibre consumption (intervention) period. Consumption of the high wheat bran fibre containing cereals led to significant improvements (largest Wilcoxon Signed Rank *p* < 0.0001) in all general wellbeing parameters. For positive/beneficial feelings (mental alertness, feeling slim, feeling happy and feeling energetic) ratings were significantly higher during the fibre intervention period than during the baseline period. Negative feelings (stress, mental tiredness, headache, feeling fat, difficulty concentrating and physical tiredness) were rated significantly lower during the fibre intervention period than during the baseline period.

Table 4 shows the mean (SD) general wellbeing feelings according to cereal intake group (Groups 1–4). Analyses showed a main effect of Group for all symptoms (Kruskal-Wallis largest *p* = 0.0305). On average, participants consuming 2 scoops or less per day (Group 1) had statistically significantly lower ratings of mental alertness and feeling slim compared to participants who consumed more than 2 scoops of cereal per day (Groups 2–4). Participants consuming more than 3 scoops of cereal per day (Group 4) rated themselves as feeling significantly happier compared to participants who consumed 2 scoops or less per day (Group 1) and more energetic than those who consumed 3 scoops or less per day (Groups 1–3).

In addition, participants consuming more than 3 scoops of cereal per day (Group 4) rated themselves as experiencing significantly less stress and as feeling less fat than those who consumed 3 scoops or less per day (Groups 1–3). Participants in Group 4 also rated themselves as experiencing less mental tiredness than those who consumed between 2 and 3 scoops per day (Groups 2 and 3).

Participants who consumed more than 2.5 scoops of cereal per day (Groups 3 and 4) experienced significantly fewer headaches than those who consumed between 2 and 2.5 scoops per day (Group 2). Interestingly, participants who consumed between 2.5 and 3 scoops per day (Group 3) reported significantly more difficulty concentrating and greater physical tiredness than those who consumed up to 2.5 scoops per day (Groups 1 and 2) and those who consumed more than 3 scoops per day (Group 4). However, adjusting for pre-treatment baseline scores, the ANCOVA analysis for physical tiredness showed there was no statistically significant differences between the 4 cereal intake groups (ANCOVA *F* = 1.14, *p* = 0.3308). The conclusions for headaches and difficulty concentrating were upheld with the adjusted analysis.

## 4. Discussion

This study has demonstrated that a dietary intervention based on regular daily consumption of one bowl of ready-to-eat breakfast cereal containing at least 5.4 g fibre (of which 70% is wheat bran fibre) for 2 weeks duration can confer significant benefits for digestive health, digestive comfort and general psychological wellbeing in habitual low-fibre consumers. In the present study, participants consumed an average total fibre intake of 13.9 g/day (of which 9.8 g per day was wheat bran fibre) from the provided breakfast cereals, over the 2-week intervention period.

Statistically significant improvements (relative to the 2 week non-intervention baseline period) were observed for most monitored digestive feelings (except wind/flatulence, indigestion and pain in the digestive system). In general, improvements in study outcomes increased with increasing cereal/wheat bran fibre consumption. However, there appeared to be an optimum daily dose of highwheat bran fibre breakfast cereal (2.5–3 scoops per day, mean intake of 14.2 g/day) for ease of defecation. The baseline prevalence of bloating in the study participants (46.5%) was commensurate with that observed in the general population [10,13] but this was reduced to 39% following the fibre intervention period. In addition, further analyses on scores of feeling bloated indicated that a greater proportion of those participants with more extreme symptoms at baseline benefited from the intervention. The results of this study provide evidence for the digestive benefits of increasing fibre intake (especially wheat bran fibre) in a representative sample. These improvements in digestive feelings occurred together with a significantly greater perceived ease of defecation and improved stool type, measured using the BSFS. Concomitant significant improvements in subjective mental alertness, feeling slim, happy and energetic, and significant reductions in subjective stress, mental tiredness, headache, feeling fat, difficulty concentrating and physical tiredness were also demonstrated. Furthermore, the reported level of improvement in digestive feelings, general wellbeing and bowel function depended upon the level of breakfast cereal/wheat bran fibre intake as indicated by the differential benefits observed between cereal intake groups (Groups 1–4). These Group differences in negative symptom relief imply a dose-response effect of wheat bran fibre on digestive health and general wellbeing. It is surprising that the significant improvement in stool type with increasing cereal/wheat bran fibre intake was not accompanied by a concomitant dose dependent increase in perceived stool quantity (Table 4). However, it is likely that stool quantity was more difficult for the participants to judge than stool type. An improvement to the study procedure would, therefore, be to include an objective measure of stool quantity in addition to self-reported stool quantity. That said, there was an overarching significant increase in perceived stool quantity during fibre the intervention period as compared to during the baseline period (Table 2). The benefits of wheat bran fibre for faecal bulking and transit time are unequivocal, and have been confirmed by EFSA health claim opinions [32]. The present study provides some insight into the concomitant benefits of increasing stool bulk and frequency. These subjective benefits include digestive feelings, general wellbeing and psychological function. These secondary benefits may, therefore, be the direct product of increases in stool bulking and stool frequency.

The physiological mechanism of action for the effect of wheat bran fibre on stool bulking and frequency is well-known, and relates to water absorption, the inability to digest cellulose [33] and wheat bran fibre morphology [34]. However, the mechanism of action for the secondary benefits to wellbeing, which are reported in this study, have not been well established. The work of Lattimore *et al.* [35] suggests that merely perceiving fibrous foods to be healthy could lead to psychological benefits such as improved mood and body shape satisfaction. In the present study it is, therefore, possible that the beliefs that the participants held about the potential health benefits of the provided breakfast cereals, or their expectations of how the breakfast cereals might impact on their general wellbeing could have affected some of the subjective study parameters.

Discussion of bowel activity is a taboo subject and presents difficulties for public health messaging. Hence less sensitive beneficial associations with fibre intake are required to increase fibre intake in the general population. Therefore, the additional benefits of increased wheat bran fibre intake demonstrated in the present study could provide a valuable messaging tool for healthcare professionals and the food industry to give motivational and appealing reasons to incorporate more fibre into the diet. 

One limitation of this study is the relatively low incidence of digestive complaints reported by the low-fibre consuming participants at baseline which limits the capacity to demonstrate large improvements in the measured digestive and wellbeing parameters. From a public health perspective, it is concerning that individuals can consume as little as 2.8 g fibre per day (participants’ baseline total fibre intake ranged from 2.8 to 15.4 g/day) and yet acknowledge no acute digestive problems in self-report measures. Without conscious awareness of digestive discomfort and the association of this with poor fibre intake, it is unlikely that the public will take action to increase their fibre intake. The possibility of under-reporting of baseline dietary fibre intake (or that the LFIQ led to an under estimation of baseline fibre intake in this sample) cannot be ruled out. The 5-point Likert scale used in the DWQ may have also contributed to this limitation. On this scale, the lowest possible response category was “none” (*i.e.*, no symptoms) which precluded a downward shift when symptoms improved, relative to normal/habitual levels, during the intervention phase. Future investigations in the area would, therefore, benefit from the development of scales which are sensitive to shifts in perceived normal symptom levels. The design of the present study could also be improved with the inclusion of a non-intervention control group who would continue with their usual diet during the intervention period.

The benefits of increasing fibre intake could be more readily shown in participants who perceive higher levels of discomfort at baseline. However, prior to commencing this study, it was decided to recruit healthy habitual low fibre consumers from the general population in order to investigate the effects of increasing fibre intake in a representative sample of the general population. If participants had been recruited on the basis of extreme symptoms of digestive problems, it is likely that these individuals would have been suffering from a specific condition such as constipation, irritable bowel syndrome (IBS), or other self-diagnosed disorders. In contrast, this study was focused on the recruitment of a representative sample from the general population to inform public health policy.

The present study suggests that there are significant and measurable acute benefits of consuming at least 5.4 g of additional fibre (3.5 g from wheat bran) per day. The benefits incorporate a breadth of outcomes, including psychological wellbeing, bowel function and digestive feelings. The reported level of improvement in digestive feelings, general wellbeing and bowel function depended upon the level of breakfast cereal/wheat bran fibre intake. Hence there may be a need to “prescribe” ideal intakes of fibre for different groups of individuals depending upon their current/habitual fibre intake. Participants who habitually consume below the recommended daily amount of fibre each day may not necessarily recognise that they have any short term problems, but they do feel the benefit of including more fibre in their diet. Self-reported bloating is observed in approximately 45% of the population and this subjective sensation can be alleviated by consumption of a minimum of 3.5 g wheat bran fibre per day. The likely mechanism for this reduction in subjective bloating is related to increased stool bulk, increased stool frequency and ease of going to the toilet.

## 5. Conclusions

The majority of the population is deficient in dietary fibre intake and positive messages, such as those provided by the results of this study, are needed to encourage increased fibre consumption. An increase in fibre intake on a population level could have considerable beneficial effects both acutely, in terms of digestive discomfort, and chronically, in terms of an array of health outcomes. The results of the present study are encouraging for both the general population without any self-perceived digestive problems and for those who experience the digestive discomfort associated with a low intake of non-fermentable fibre (e.g., wheat bran fibre) containing foods.
